# The Belgian Field Hospital from within

**Published:** 1915-03-20

**Authors:** 


### THE BELGIAN FIELD HOSPITAL FROM WITHIN.

### The Surgeon-in-Chief's Experiences.

The urgent claims of the war have carried many British surgeons into new lands and have provided them with new experiences. Hardly less than the soldier, the surgeon has had to accommodate himself to the practice of his art under novel conditions, and while in each case principles may have remained steadfast, fresh lessons have had to be learned on numerous points of detail. The time will doubtless come when these lessons will be formulated by experts alike in the department of surgery as in those of tactics and strategy. But that time is not yet, and we must be content to wait with the confident assurance that both successes and failures are being duly noted as the necessary basis on which fresh accommodations can be safely adjusted. There are, however, aspects of the war other than those which pertain to technical criticism, and for news of these it is hard to wait with patience. So far as silence is a military necessity, patience must needs be practised; yet very naturally we long for the human touch, and desire to know what individuals have felt and seen and done and suffered. Hence the large demand, widely recognised in the newspapers, for letters from men who have actually experienced the stress of the conflict and have seen with their own eyes the widespread consequences of the stricken field. Whether soldiers or surgeons, they still are men, and therefore their voices touch a chord which finds an answering note in all our hearts. Time and opportunity are wanting to many who must have much to say, and even when these are provided the appropriate word is not open to all. All the more welcome, therefore, is a book in which all the necessary conditions exist, and wherein the experienced and competent observer and the ready writer are fused in one and the same person for our common information and benefit.

The above remarks are prompted by a book,* entitled simply "A Surgeon in Belgium," by Mr. H. S. Souttar. Possibly such a title may suggest a technical treatise and the activities of the operating theatre. But the reader may at once be reassured on this point. That the Surgeon-in-Chief of the Belgian Field Hospital could write a book about the war with surgery excluded would be a vain expectation, and, indeed, in their broader aspects hospital experiences and activities take here the prominent place which is their due. But the chief interest of Mr. Souttar's volume arises not from its technical qualities, but from much wider values. It is written by one who, having the opportunities of the military surgeon, possesses at the same time the eye of the sympathetic observer, a saving sense of humour, and the happy pen of the literary artist. In a word, the book is essentially a picture of actual happenings in the stress of acute and urgent responsibilities. While reading its pages we can see and feel the pressure of events and are conscious of the common-sense and courage with which emergencies were met, the cheerful goodwill of men and women under trial, and the sense of tears in human lives. Names which in military dispatches, and even in newspaper articles, are little more than geographical expressions, take on a new quality when we are made to realise them as the scenes of events in which the writer and his colleagues—men of like passions with ourselves—met unusual experiences with what reads like easy common-sense and good temper. This, indeed, is the supreme quality of the book: that it tells us not of the wonderful achievements of some great personage, but rather of the sensible and cheerful doings and happy dispositions of a number of colleagues among whom, had fortune been kind, we ourselves might perhaps have been numbered. To remove wounded in London omnibuses, marked as destined for Hendon, during a bombardment, to eat bacon cooked by a lady who writes excellent novels, to witness the desolation of a country and of a nation, to practise surgery amidst the stress of a siege, to see a great cruiser sunk by the infernal artifice of a submarine, and to eat an excellent salad, only to discover too late that it had been made with castor oil—these are experiences which few surgeons compass in a lifetime, much less in the course of a few months. To still fewer is bestowed the happy ability to see all such events steadily and in due proportion, and to make of them a story of moving human interest in which all our sympathies must needs be engaged.

To the strictly surgical portions of Mr. Souttar's book we shall hope to make some reference on a future occasion. Our more immediate object is to recognise its appeal as a chronicle and description of war experiences, and to congratulate a *confrère* on the exceptional use he has made of exceptional opportunities. Members of the medical profession are, of course, particularly qualified to appreciate his book, as they can in some degree measure the burden of responsibility which rested on the writer while he yet managed to see things other than his immediate duties. The book, however, is for a wide audience. It recites many moving incidents by flood and field. Neither on its surgical nor on its military side does it attempt the formal or the precise. But the message of the war is vivid enough, though it is not announced by statistics or displayed in maps. The thrill and stimulus as well as the terror and wickedness come home to the reader because of the unpretending art with which the story is told. Those who complain, perhaps justly, that the war in its human incidents is too closely curtained by an official fog can surely find in its pages an opportunity to realise both the horrors and pathos of the occasion, and will see in action the qualities by which men and women of the right temper raise themselves, all unconsciously, to the height of the supreme argument.

* *A Surgeon in Belgium.* By H. S. Souttar, F.R.C.S. London: Edward Arnold. 1915. Price 8s. 6d. net.

